# Giant Cell Arteritis with Intracranial and Extracranial Aneurysms: A Case Report and Brief Review of the Literature

**DOI:** 10.7759/cureus.62395

**Published:** 2024-06-14

**Authors:** Varsha Prasad, Nick Tarlov, Amanda Frugoli, Angelica Shepard

**Affiliations:** 1 Internal Medicine, Community Memorial Hospital, Ventura, USA; 2 Interventional Neurology, Community Memorial Hospital, Ventura, USA; 3 Graduate Medical Education/Internal Medicine, Community Memorial Hospital, Ventura, USA; 4 Graduate Medical Education/Rheumatology, Community Memorial Hospital, Ventura, USA

**Keywords:** conventional cerebral angiogram, giant cell arteritis (gca), temporal artertitis, internal carotid aneurysm, intracranial aneurysm

## Abstract

Giant cell arteritis (GCA) is a relatively rare, auto-immune vasculitis, more common in women over age 50. It is important to recognize and treat the disease early to prevent late complications of permanent vision loss. Inflammation-associated weakening of vessel walls involved by GCA may also represent a potential etiology for intracranial aneurysm development. In this report, we describe an atypical presentation of GCA confirmed with temporal artery biopsy with associated manifestations including intracranial right posterior communicating artery aneurysm and extracranial right internal carotid aneurysm. Our patient in a 78-year-old female who presented with progressively worsening headaches that began 10 days prior to admission. These were described as global, non-pulsatile, and located over her occiput. She reported associated jaw soreness while chewing or claudication. Her erythrocyte sedimentation rate (ESR) was elevated at 74 mm/hr. Magnetic resonance angiogram showed a right posterior communicating artery aneurysm measuring 5 mm and a right cervical carotid lengthwise dissecting aneurysm measuring 12 mm. Left temporal artery biopsy confirmed the diagnosis of GCA. High-dose steroid therapy was initiated and was continued for treatment of GCA with resolution of symptoms at her one month follow-up. This case highlights a rare instance of cervical internal carotid aneurysm and intracranial aneurysm associated with GCA, emphasizing the systemic nature of this vasculitis.

## Introduction

Temporal arteritis (TA) has had an updated nomenclature to giant cell arteritis (GCA). It remains a relatively rare systemic vasculitis of medium to large-sized vessels that more likely affects women aged over 50 years [1,2]. As the prior name suggests there is a predisposition to affect the cranial arteries, specifically the temporal artery. It classically presents with headaches, jaw claudication, tenderness over the temporal arteries, and elevation of inflammatory markers but in late or severe cases, it can be associated with irreversible vision loss as the short posterior ciliary arteries or central retinal artery may be involved [1,2]. Other symptoms of vascular irregularities may manifest as limb claudication, discordant blood pressures, abnormal radial pulse, and temporal artery abnormalities [2]. It is also associated with polymyalgia rheumatica [3]. Recently, the 1990 American College of Rheumatology criteria was updated to include additional systemic symptoms associated with the disease and advances in non-invasive vascular imaging that included large vessels outside of the cranium [1,3]. The overall diagnostic criteria were changed from three out of five criteria to a point system with added features (see Appendices). 

GCA is typically described in two settings: GCA of the temporal artery and GCA causing aortic root disease [1,3]. Approximately 10-30% of patients with GCA demonstrate aortic root dilation [3-5]. There are very few reports existing that document histologic evidence of aortic root disease in patients with GCA of the temporal artery [3,4]. However, these two entities are generally accepted as representing parts of a spectrum. GCA of the temporal artery classically includes medial chronic inflammation of medium to large arteries with giant cells engulfing fragments of internal elastic lamina [6,7]. Prominent inflammation of muscular vessel walls may also cause luminal stenosis and ischemia, as seen with disease involvement of the ophthalmic artery and posterior ciliary branch with ischemia-associated vision loss [8]. Inflammation-associated weakening of vessel walls involved by GCA may also represent a potential etiology for intracranial aneurysm development [9]. Multiple foci of intracranial arterial dissection have been reported as a possible uncommon presentation of GCA; however, intracranial arteries are not typically involved in cases of GCA [9].

In this report, we discuss a previously healthy female who was diagnosed with GCA with supporting presentation and confirmatory temporal artery biopsy and was found to have intra-and extracranial aneurysms on neuroimaging. We highlight the systemic nature of this vasculitis and describe rare instances of a cervical internal carotid and intracranial aneurysms. 

## Case presentation

A 78-year-old right-handed female presented with daily headaches which began 10 days prior to admission. She reported a gradually progressive headache over three hours associated with diarrhea and nausea. The persistent headaches were global, non-pulsatile, and localized over her occiput. Over-the-counter analgesics provided only minimal relief. She described associated jaw soreness while chewing and reported no previous similar episodes of headache and no personal or family history of migraines. 

**Figure 1:**
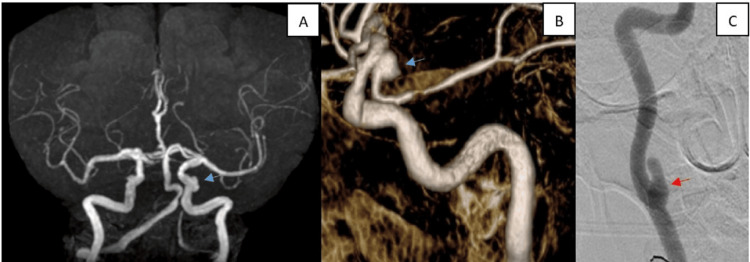
(A) Magnetic resonance angiogram and (B) a conventional cerebral angiogram reconstruction confirmed the presence of a wide-necked right posterior communicating artery aneurysm approximately 5 mm in size (blue arrow). (C) Right cervical carotid aneurysm (possible pseudoaneurysm) measuring 12 mm in its greatest dimension (red arrow)

She was afebrile on admission. Her erythrocyte sedimentation rate (ESR) was elevated at 74 mm/hr (reference: <30 mm/hr). A magnetic resonance angiogram (MRA) imaging study revealed a saccular aneurysm at the origin of the right posterior communicating artery and an aneurysm (possible pseudoaneurysm) of the right cervical internal carotid artery (Figure 1). These findings were confirmed with a conventional cerebral angiogram which revealed a wide-necked right posterior communicating artery aneurysm approximately 5 mm in size and a right cervical carotid aneurysm (possible pseudoaneurysm) measuring 12 mm in its greatest dimension. 

**Figure 2:**
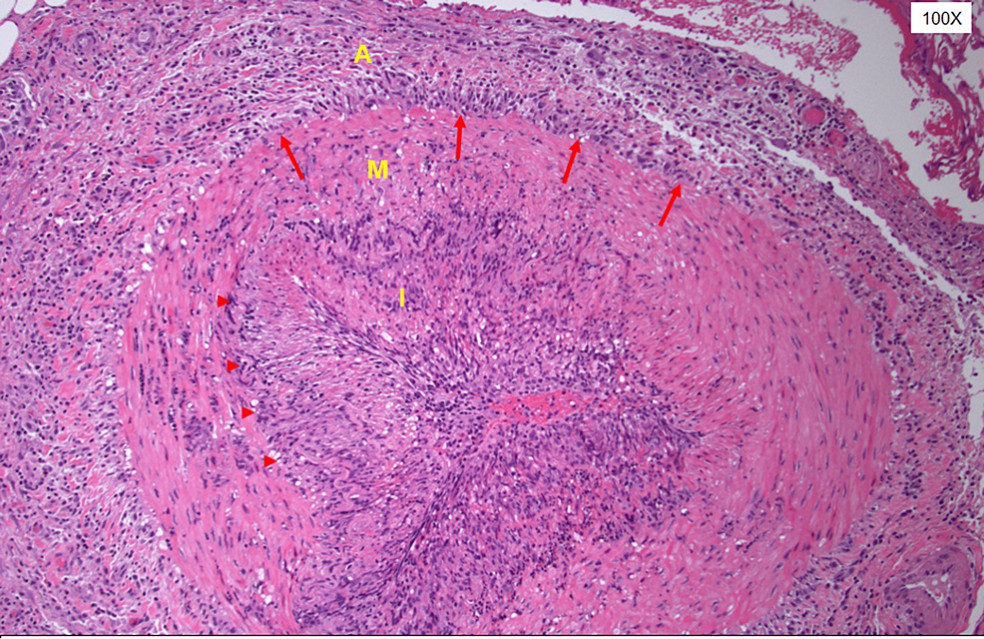
Biopsy of the left temporal artery revealed prominent lymphoplasmacytic inflammation with numerous histiocytes involving the internal and external elastic lamina (highlighted by red arrows)

**Figure 3:**
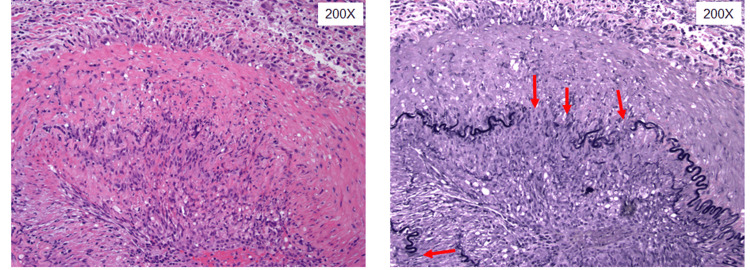
Histopathology of the left temporal artery demonstrating elastic stain demonstrated patchy destruction of the internal elastic lamina on 200X powered field and prominent lymphoplasmacytic inflammation with numerous histiocytes involving the internal and external elastic lamina (left) and elastic stain demonstrated patchy destruction of the internal elastic lamina (highlighted in black arrows).

Given her new headache, jaw claudication, and elevated ESR, temporal arteritis was considered. She presented with four of the European Alliance of Associations for Rheumatology (EULAR) diagnostic criteria. She was started on high-dose corticosteroids 1 mg/kg prior to obtaining a temporal artery biopsy. A biopsy of the left temporal artery revealed prominent lymphoplasmacytic inflammation with numerous histiocytes involving the internal and external elastic lamina (Figure 2). Elastic stain demonstrated patchy destruction of the internal elastic lamina (Figure 3). These biopsy findings were consistent with the diagnosis of GCA. 

A day following steroid initiation, the patient reported improvement in her headaches. She was discharged home on prednisone 55 mg oral daily with detailed discharge instructions including close follow-up with plans for extended taper with rheumatology. 

At the patient’s one-month follow-up, her headaches had completely resolved and the jaw claudication had markedly improved. Since the aneurysm was determined to not be a significant risk for rupture, the patient decided she would like to monitor her aneurysms without intervention. She was planned to have follow-up neuroimaging in six months to determine size and growth. 

## Discussion

GCA of the temporal artery presents with a variety of symptoms including headache, jaw claudication, and, rarely, cerebrovascular events such as TIA or stroke [4]. Multiple intracranial aneurysms may represent an additional rare manifestation of GCA [10]. Aneurysms are typically associated with weakened muscular vessel walls due to a variety of etiologies including congenital defects, genetic predisposition, atherosclerosis, trauma, infection, or other causes of vasculitis. Pseudoaneurysms may also arise in association with vascular injury leading to micro-ruptures of the vessel wall and associated leakage of blood into the surrounding tissue [10]. This can have a similar appearance to a true aneurysm in imaging studies. 

Histopathologic review of a temporal artery biopsy confirms the diagnosis of GCA of the temporal artery. Typical histologic sections of an involved temporal artery show small and medium-sized arteries with inflammation composed of transmural and perivascular lymphocytic cells with histiocytes and associated destruction of the vessel wall internal elastic lamina (seen as fragmentation on elastic silver-stained sections) [6,7]. Multinucleated giant cells and granulomatous inflammation are also present in approximately 50% of biopsies; however, lymphohistiocytic inflammation of the vessel alone (without giant cells or granulomas) is sufficient for diagnosis [6]. In the setting of empiric steroid treatment prior to biopsy, it is generally accepted that treatment will not affect the rate of positive biopsies for up to 10-14 days after initiation [11]. However, due to the patchy nature of vessel involvement by inflammation, a temporal artery biopsy may not always be diagnostic. 

GCA is primarily associated with inflammation (of unknown etiology) involving extracranial arteries, specifically the temporal artery. Involvement of intracranial arteries is rare as it is hypothesized by some that GCA may involve an autoimmune reaction against arterial elastic tissue, which is significantly reduced within the first 5 mm of entering the intracranial space [12]. Following this hypothesis, one could suggest that any portion of the extradural arterial circulation within the head and neck may be involved [8]. The intracranial arteries have thin walls with significantly reduced elastic fibers in the media and adventitia and absent vasa vasorum, which provide support to the walls of larger extradural arteries [12]. 

Involvement of intracranial/intradural arteries in patients with GCA of the temporal artery is a rare complication and appears to represent a subset of patients with a potentially fatal course that may be resistant to corticosteroid therapy [12,13]. In a case report by Parra and colleagues, a 56-year-old man with biopsy-proven GCA of the temporal artery was also found to have extensive involvement of intracranial arteries, including dissection of the left vertebral and bilateral internal carotid arteries by imaging studies [9]. Another case report describes GCA of the temporal artery with vertebral artery dissection, occurring secondary to chronic arteritis, with autopsy histology supporting GCA involvement of the vertebral artery [14]. In a case series by Siemonsen et al, imaging demonstrated vertebral artery involvement in four patients positive for GCA on temporal artery biopsy [15]. Collectively, the literature suggests that GCA of the temporal artery can occasionally involve intracranial arteries with potentially fatal complications.

Similar to the case reported here, most of the above-referenced studies utilize imaging findings as evidence suggestive of intracranial involvement in patients with GCA of the temporal artery. However, a few reports, including one by Sheehan et al., describe post-mortem histology supporting the involvement of intracranial arteries [14]. In their report, they illustrate post-mortem histopathology of the intracranial left vertebral artery showing dissection of the muscular wall with granulomatous inflammation, multinucleated giant cells, and disruption of the internal elastic lamina [14]. Another report describes an exceedingly rare case of a 19-year-old woman who died of subarachnoid hemorrhage associated with rupture of isolated intracranial vertebral and basilar artery aneurysms with post-mortem histology consistent with GCA in the absence of temporal artery involvement [16]. 

In a case series by Säve-Söderbergh et al., GCA was identified as the cause of death, secondary to arteritis affecting various large and medium vessels [17]. Of the nine patients, two died of myocardial infarction caused by GCA in the coronary arteries, another two died of dissecting aneurysms of the aorta, and the remaining five patients died of cerebral stroke secondary to GCA. As mentioned earlier, GCA of the aortic root and the temporal artery are thought to represent manifestations of a spectrum of disease in GCA. Similarly, other rare foci of involvement may also be considered in this spectrum. 

A retrospective medical record review by Sanchez-Alvarez analyzed the rare intracranial involvement in GCA [18]. This showed most frequently affected intracranial arteries were the internal carotid artery (100%), vertebral artery (67%), posterior cerebral artery (33%), and posterior inferior cerebral artery (11%) [18]. In a prospective assessment of intracranial vasculitis, intradural involvement was seen in at least 50% of patients positive for GCA [18]. Additionally, mural inflammation of the vertebral arteries was observed in four patients positive for GCA and the proximal MCA in one patient [15]. However, it is possible that the frequency of intracranial GCA is underestimated due to a selection bias in patients with GCA who undergo CNS imaging [15]. When cerebral aneurysms are located in unusual sites, GCA should be considered.

Our patient presented with typical symptoms of GCA with headache, jaw claudication, age over 50, and elevated ESR. Biopsy of the temporal artery did not show giant cells, but giant cells are seen in only 50% of biopsies [10]. Her temporal artery had a typical pattern of lymphohistiocytic inflammation of the temporal arterial wall which was sufficient to make the diagnosis of temporal arteritis [2]. There are limited reports of patients with intradural aneurysms who were suspected to be resultant of GCA in the literature [2-4,7-9]. However, the term GCA has in these cases been used to describe a disease in children and young adults that is distinct from the TA that occurs in the elderly because of the age at which it presents and because, in many cases, there is a lack of the lack of systemic inflammation in children and young adults.

Despite being a straightforward vignette of GCA, this case identified a posterior communicating artery aneurysm and extracranial carotid aneurysm. There are reports of two previously healthy teenage women who died of ruptured posterior circulation aneurysms and giant cells were seen in the aneurysm wall [1,8]. In a similar case, a 22-year-old female presented in Japan with a ruptured fusiform posterior inferior cerebellar artery aneurysm whose wall was infiltrated with giant cells [3]. The authors did not describe the presence of any systemic inflammatory disease, and named her disease “primary GCA of the central nervous system” or “granulomatous angitis of the central nervous system.” 

The current presentation was different than the above with elderly age, and symptomatic presentation was consistent with temporal arteritis. Our patient had no prior neuroimaging and it is possible that the right posterior communicating aneurysm was an incidental finding and not the result of GCA. However, she did not have traditional risk factors for the formation of aneurysms of the circle of Willis such as smoking or hypertension. Since she was found to have an inflammatory disease, possibly the aneurysm and inflammatory disease are related. 

Our patient also had an aneurysm of the cervical internal carotid artery, which is much more unusual than her intracranial aneurysm. Takasayu arteritis, which can involve the cervical internal carotid artery is not likely because of the age of our patient at the time of presentation, and the presence of inflammation characteristic of TA in her temporal artery. Only one case has been reported of temporal arteritis and fusiform aneurysms of common carotid (2.6 cm), innominate (4 cm), and subclavian arteries (2.5 cm). The patient was 82 years old and had classic symptoms of GCA which was proven by a biopsy of the temporal artery [19]. The limited case reports and our case may suggest that GCA may cause some tendency for aneurysm formation in the cervical arteries. 

This case report captures a rare occurrence of intra and extracranial aneurysms that could be associated with GCA and highlights that this vasculitis has systemic involvement. 

## Conclusions

This case demonstrates a rare case of GCA of the temporal artery with associated findings including aneurysms of the intracranial right posterior communicating and extracranial right internal carotid arteries. Overall, GCA of the temporal artery represents one of the more common of a variety of rare presentations, which may be seen in the spectrum of potential rare entities encompassed by GCA. Despite the uncommon nature of disease, it is important to be aware of potentially fatal complications including intracranial artery involvement with associated aneurysms.

## Appendices

**Table 1:** Comparison of the 1990 and updated 2022 ACR and EULAR criteria for GCA References: Hunder et al., 1990 [1], Ponte et al., 2022 [2] ACR: American College of Rheumatology; EULAR: European Alliance of Associations for Rheumatology; GCA: giant cell arteritis; ESR: erythrocyte sedimentation rate; FDG: fludeoxyglucose F18

	1990 ACR Criteria	2022 ACR/EULAR
Age at onset > 50 years old	Yes, part of the criteria	Absolute requirement
New Headache	Yes, part of the criteria	Part of the point system (2+) and recommend temporal headache
Temporal Artery Abnormality as defined by “Temporal artery tenderness to palpation of decreased pulsation”	Yes, part of the criteria	Part of the point system (2+)
Elevated ESR as defined by “>50 mm/hour by the Westerngreen method”	Yes, part of the criteria	Elevated SED using 1990 criteria Or CRP > 10mg/liter^2^
Abnormal Artery Biopsy as defined by “Biopsy specimen with artery showing vasculitis characterized by a predominate of mononuclear cell infiltration OR granulomatous inflammation with multinucleated giant cells”	Yes, part of the criteria	Biopsy with 1990 criteria Or halo sign on temporal artery ultrasound
Jaw or Tongue Claudication	Not part of the 1990 criteria	Added in the 2022 criteria (2+)
Sudden Vision loss	Not part of the 1990 criteria	Added in the 2022 criteria (3+)
Morning Stiffness in Shoulder/Neck	Not part of the 1990 criteria	Added in the 2022 criteria (2+)
Bilateral Axillary Involvement	Not part of the 1990 criteria	Added in the 2022 criteria (2+)
FDG-PET Activity Throughout the Aorta	Not part of the 1990 criteria	Added in the 2022 criteria (2+)
Criteria	3/5 Criteria need to be present	>6 points needed for the classification
